# Pathogenesis of Thromboembolism and Endovascular Management

**DOI:** 10.1155/2017/3039713

**Published:** 2017-01-05

**Authors:** Sasan Behravesh, Peter Hoang, Alisha Nanda, Alex Wallace, Rahul A. Sheth, Amy R. Deipolyi, Adnan Memic, Sailendra Naidu, Rahmi Oklu

**Affiliations:** ^1^Division of Vascular & Interventional Radiology, Mayo Clinic Arizona, 5777 E Mayo Blvd, Phoenix, AZ 85054, USA; ^2^Department of Radiology, Mayo Clinic Arizona, 5777 E Mayo Blvd, Phoenix, AZ 85054, USA; ^3^Department of Interventional Radiology, Division of Diagnostic Imaging, MD Anderson Cancer Center, 1515 Holcombe Blvd, Houston, TX 77030, USA; ^4^Division of Interventional Radiology, Memorial Sloan Kettering Cancer Center, 1275 York Ave, New York, NY 10065, USA; ^5^Center of Nanotechnology, King Abdulaziz University, Jeddah 21589, Saudi Arabia

## Abstract

Venous thromboembolism (VTE), a disease that includes deep venous thrombosis (DVT) and pulmonary embolism (PE), is associated with high mortality, morbidity, and costs. It can result in long-term complications that include postthrombotic syndrome (PTS) adding to its morbidity. VTE affects 1/1000 patients, costs $13.5 billion annually to treat, and claims 100,000 lives annually in the US. The current standard of care for VTE is anticoagulation, though thrombolysis may be performed in patients with PE and threatened limb. This review discusses pathogenesis and medical treatment of VTE and then focuses on endovascular treatment modalities. Mechanical- and catheter-directed thrombolysis (CDT) is discussed, as well as patient selection criteria, and complications. The first prospective study (CaVenT) comparing CDT with anticoagulation alone in acute DVT, despite study shortcomings, corroborates the existing literature indicating improved outcomes with CDT. The potential of the ongoing prospective, multicenter, randomized ATTRACT trial is also highlighted.

## 1. Introduction

Venous thromboembolism (VTE) is a disease process most commonly manifested as deep vein thrombosis (DVT) and/or pulmonary embolism (PE) that impacts approximately 1 out of every 1000 patients [1]. The clinical ramifications of VTE include both acute sequelae such as sudden death and complications of anticoagulation and chronic sequelae such as postthrombotic syndrome (PTS) and chronic thromboembolic pulmonary hypertension (CTEPH) [2, 3]. The estimated total US expense associated with VTE is between $13.5 and $69.5 billion. Additional nonmedical costs include lifestyle modifications, caregiver expenses, and cost of life lost [3, 4]. Venous thrombosis can be treated with systemic and endovascular approaches in an effort to improve the 5% all-cause mortality within 1 year attributed to VTE [2]. In this review, we summarize the risk factors, pathogenesis, complications, diagnostic criteria and tools, and medical and endovascular management for VTE.

## 2. Venous Thromboembolism

### 2.1. Epidemiology

The current incidence of venous thrombosis and thromboembolism is approximately 1 per 1,000 adults annually. One-third of patients present with PE, while the remainder present with DVT. The 1-month mortality is as high as 6% with DVTs and 10% with PEs, though postmortem studies suggest that these already high mortality rates are likely underestimates. Autopsy results estimated the mortality to be as high as 30%, predicated on the observation that many PEs are not diagnosed at the time of death [5]. Moreover, hypercoagulable states such as malignancy increase the rate of mortality with PE and DVT when compared with idiopathic causes.

Venous thromboses are highly morbid. For patients that develop DVTs, the risk of recurrence is approximately 7% despite anticoagulation (AC) therapy [6]. Beyond the acute complications and despite timely initiation of anticoagulation, DVTs can lead to persistent chronic disease that can be severely disabling. The constellation of chronic symptoms caused by impaired venous return is called postthrombotic syndrome (PTS) and occurs in up to 20–50% of patients following an acute DVT [7, 8]. PE can also have devastating chronic sequelae termed chronic thromboembolic pulmonary hypertension (CTEPH). Although the exact costs are difficult to quantify, it is thought that both clinic entities greatly increase the cost of venous thrombosis [9].

### 2.2. Pathogenesis

The German physician Rudolf Virchow described three factors that contribute to the development of VTE, comprising Virchow's triad: stasis, vessel damage, and a hypercoagulable state [14]. Beyond postsurgical and trauma-related cases, stasis may play the largest role in the development of venous thrombosis [15]. The development of venous thrombosis begins at the valves or venous sinuses [16–18]. Venography studies have shown that contrast media can linger in these areas for up to 27 minutes following administration [19]. Autopsy studies confirm these locations to be the most frequent sites of thrombosis initiation [20]. Venous thrombosis originates as small fibrin deposits in these areas of low flow. The areas of deposits then grow by apposition to occlude vessels and eventually trigger the coagulation cascades. Similarly, postsurgical or trauma-related endothelial injury can also trigger this fibrin nidus [16, 21]. Antithrombotic proteins such as thrombomodulin and endothelial protein C receptor (EPCR) are regionally expressed on the valves and are sensitive to hypoxia and inflammation. Stasis at the valvular sinus has been linked to hypoxia and increased hematocrit forming a hypercoagulable microenvironment. These conditions including acute inflammation lead to downregulation of the aforementioned proteins and thereby promote the formation of thrombus. Hypoxia can also lead to the upregulation of procoagulants such as tissue factor on endothelium and P-selectin (an adhesion molecule) also on endothelium leading to recruitment of leukocytes or monocyte derived leukocyte microparticles also containing tissue factor. Tissue factor is considered the initiator of coagulation and in concert with P-selectin are essential components of thrombosis [22]. Without sufficient flow, the fibrin deposits activate clotting factors locally; blood coagulation inhibitors are consumed without the influx of new inhibitors. An anticoagulant pathway such as the protein C pathway, which leads to the inactivation of cofactors Va and VIIIa, is triggered by EPCR and thrombin bound to thrombomodulin. Tissue factor initiated coagulation is inhibited by tissue factor inhibitor. Thrombin, a coagulation enzyme, is blocked by antithrombin which in turn is stimulated by heparin-like proteoglycans [22]. As the coagulation cascade unfolds, fibrin, red blood cells, and platelets form the intravascular deposit known as the venous thrombus [23]. The venous clot is described as being made of two regions: the red cell rich fibrin clot parallel to the endothelium and lines of platelet rich white thrombus commonly referred to as the lines of Zahn within the clot separating regions of red thrombus. Genetic variants such as high levels of coagulation factor VIII, von Willebrand factor, factor VII, and prothrombin are all linked to an elevated risk of thrombus formation. Most commonly, a defect in factor V Leiden, which usually ensures factor Va resistance to activated protein C, is found in 5% of Caucasians [22]. Other risk factors attenuate genetic propensity to clot formation including presence of lupus anticoagulants and use of oral contraception; cancer can block blood flow, lead to increased tissue factor which initiates coagulation, and lead to the release of procoagulant lipid microparticles. Venous valves are impaired and vessels are prone to stasis with increasing age. Increased levels of coagulation factor are seen with decreases in the efficacy of natural anticoagulants and immobilization and risk of infection is more commonplace [22]. The “multiple hit hypothesis” explains that while venous stasis is the dominant contributor to venous thrombosis development, it is seldom the sole contributor to clot formation [22]. Clinically and experimentally, it is now appreciated that at least two of the three Virchow's triad are needed for clinically significant venous thrombosis to form. Animal models have shown that venous flow alterations alone are insufficient to produce thrombus [24]. Numerous retrospective reviews of venous thrombosis patients reveal that the majority of patients have multiple risk factors [25].

### 2.3. Risk Assessment and Diagnosis

Symptom recognition is crucial for early diagnosis of DVT and PE. Increased suspicion is prompted by risk factors such as coagulopathies, advanced age, cancer, antiphospholipid syndrome, infection, inflammatory disorders, nephrotic syndrome, immobilization, obesity, hormonal therapy, and pregnancy. DVT classically presents with calf pain, thigh pain, or cramping. PE is a more challenging diagnosis, given its variable presentation and severity; typical symptoms of dyspnea, presyncope, syncope, and pleuritic pain overlap with numerous other clinical entities. Suspected PE management is dependent on risk stratification. Wells' or Geneva score can be used to risk-stratify patients. The Geneva score assesses PE with parameters such as age, pulse, and hemoptysis.

In patients that are considered to be of low risk, the Pulmonary Embolism Rule-out Criteria (PERC) can be used to determine whether further workup is necessary. PERC can swiftly be calculated without invasive testing, and if PERC rules out PE, the likelihood of PE is very low. A positive PERC is followed by a D-dimer assay. As in the evaluation for DVT, a normal D-dimer renders PE very unlikely despite a high pretest probability. Moderate risk of PE should be followed by a high sensitivity D-dimer, and if abnormal, the clinician should proceed with CT angiography. High risk of PE should promptly be assessed with CT angiography, bypassing all other tests. Those with contraindications to contrast can receive a ventilation perfusion (VQ) scan in lieu of CT angiography [26].

Wells' criteria are also widely used to assess DVT likelihood. Wells' criteria include extremity edema, tenderness, and cancer diagnosis. For patients determined to be of low or moderate suspicion for DVT, a D-dimer assay is often performed. A normal D-dimer in low or moderate risk patients can confidently exclude DVT. If the D-dimer is abnormal at any level of risk, duplex ultrasonography is indicated. All high-risk patients may receive a diagnostic ultrasound (US) in addition to a D-dimer assay. Positive ultrasonography for DVT leads to treatment, whereas a negative ultrasound in a high-risk patient warrants repeat ultrasound in 7 days [26].

## 3. Clinical Outcomes of VTE

### 3.1. Pulmonary Embolism (PE)

Clinical outcomes for patients with acute PE vary greatly [27]. To facilitate decision-making in this unpredictable clinical setting, multiple specialty groups and societies have established recommendations regarding the risk stratification and management of PE. However, several of these guidelines employ idiosyncratic classification systems, causing unnecessary confusion for clinicians seeking guidance. Fundamentally, the principal discrepancies involve the definition for patients at “intermediate risk,” also described as patients with “submassive PE.” Overall, definitions for “high risk” (also known as “massive PE”) and “low risk” (also known as “nonmassive PE”) are for the most part consistent.

In a 2011 statement, the American Heart Association (AHA) defined massive PE as patients with sustained hemodynamic instability [27]. Hemodynamically stable patients who have risk factors for impending instability (right ventricular dysfunction, elevated brain natriuretic peptide, or myocardial necrosis) are categorized as submassive PE. Patients without hemodynamic instability and the above risk factors are classified as low risk. The guidelines recommend therapeutic anticoagulation for all patients with PE and no contraindication. The use of thrombolytics is not directly endorsed for any classification, though their use is suggested for massive PE patients and may be considered for submassive PE patients.

Unlike the AHA, the American College of Chest Physicians (ACCP) guidelines do not define discrete categories for PE [28]. However, similar to the AHA, the ACCP guidelines are circumspect on the use of thrombolytics, directly recommending that thrombolytics* not* be used unless patients present with hemodynamic instability.

On the other hand, the European Society of Cardiology (ESC) defines a four-tier classification system for PE: low risk, intermediate-low risk, intermediate-high risk, and high risk [29]. These guidelines use the PESI score to define the intermediate risk strata. The presence or absence of right ventricular dysfunction and myocardial necrosis then subclassifies patients into intermediate-high or intermediate-low categories. The ESC guidelines are more aggressive than the AHA or ACCP guidelines regarding the use of thrombolytics: thrombolytic use is directly recommended for patients in the high-risk category and can be considered for intermediate-high-risk patients.

### 3.2. Postthrombotic Syndrome

Postthrombotic syndrome (PTS) is a debilitating chronic outcome of proximal DVT, which is a chronic clinical phenomenon [30, 31]. 20 to 50% of patients who have a proximal DVT will suffer from postthrombotic syndrome within 2 years [32]. It has been suggested that PTS is due to incomplete recanalization or and/or permanent damage to the venous valves resulting in valvular reflux [31]. Its pathophysiology is not well understood, but, clinically, PTS manifests itself as leg heaviness, fatigue, aching, and edema [32]. Severe PTS, found in 3% of patients after suffering a DVT, additionally presents with venous ulcers [32]. The symptomatology may be exacerbated or confused by comorbid conditions that the patients may have including congestive heart failure, lymphedema, obesity, obstructive sleep apnea, diabetic complications, and peripheral vascular disease [30]. Persistence and severity of the syndrome at one month are associated with worse prognosis over the next two years. The Villalta grading scale has been implemented to standardize and score PTS. Pain, edema, erythema, induration, changes in skin color, and venous ectasia are scored by clinicians from 0 to 3, with three being the most severe. A score of 5 or more is indicative of PTS [32]. Preventing venous thrombosis is the best way to prevent PTS. However, after the initial insult, AC regimens have been largely ineffective in reducing the morbidity resulting from PTS. Strides have been made in the past decades to achieve therapeutic INR levels with warfarin after DVT as well as other novel oral anticoagulant agents [31]. Common medical therapies include LMWH, intravenous unfractionated heparin, subcutaneous unfractionated heparin, and warfarin. Along with lifestyle modifications, elastic compression stockings are also commonly used in PTS treatment, although their effectiveness, as well as the ideal degree of compression, is controversial [31, 33]. To address the suggested PTS pathophysiology of retained thrombosis, catheter-directed thrombolysis has also been used in treatment to prevent PTS. PTS incidence has declined, but a concomitant improvement in quality of life has not been demonstrated as yet [31].

## 4. Medical Management of DVT and PE

Medical management is generally the first line of therapy for DVT and PE. Thrombolytic therapy is indicated only in cases of a massive PE or extensive DVT [26]. Otherwise, intravenous unfractionated heparin, subcutaneous low molecular weight heparin (LMWH), and fondaparinux are often given in the acute phase of DVT or PE [2, 26]. Transition to a vitamin K antagonist, such as warfarin, dosed to a therapeutic INR of 2-3, follows in the short and long term [26, 33]. The disadvantages of subcutaneous medication administration with LMWH and frequent follow-ups at a warfarin clinic are partly responsible for the advent of direct oral anticoagulants (DOACs). These medications are not routinely monitored with blood tests and are associated with fewer drug-drug interactions; however DOACs lack the long-term data available for vitamin K antagonists and LMWH [2, 26]. The DOACs that are approved for venous thrombosis management in the US include rivaroxaban, apixaban, edoxaban, and dabigatran. In a study comparing the DOACs, apixaban had a lower risk of critically relevant nonmajor bleeding. When compared to the standard of care of LMWH and warfarin, apixaban and rivaroxaban were associated with fewer major bleeding instances [2]. Both are alternatives to LWMH and warfarin in acute and short-term treatment. Dabigatran, a direct thrombin inhibitor, was associated with increased gastrointestinal bleeding and myocardial infarction in older patients when compared to warfarin; however, it may be a reasonable alternative to warfarin in the short term [26]. At least three months of anticoagulation therapy is recommended after venous thromboembolism [26, 33]. Recent guidelines advise that pregnancy associated VTE should be treated with anticoagulation therapy for the duration of the pregnancy and up to 6–12 weeks postpartum, for a minimum duration of at least 3 months in total. Furthermore, patients should be considered for thromboprophylaxis in any future pregnancies [26, 34, 35]. Long-term anticoagulation can be achieved with the same medications, or low dose aspirin can be implemented for those who are not candidates for long-term AC [26, 34, 35]. Overall, the goal of therapy is to prevent recurrence all the while minimizing risks of bleeding. High systemic levels of AC therapy can lead to severe bleeding outcomes with high morbidity and mortality. A study comparing the case-fatality rate and major bleeding with AC after venous thrombosis showed decreased risk of VTE recurrence over time, but bleeding risks remain stable [36].

The decision to pursue inpatient versus outpatient AC treatment should integrate the patient's overall health, accessibility to medical care, and support at home. In the case of PE, echocardiography and cardiac biomarkers can suggest mortality estimates, affecting the choice of treatment setting. The HESTIA criteria and the simplified Pulmonary Embolism Severity Index (sPESI) are validated resources in assessing outcomes and aid in clinical decision-making [26].

Residual vein thrombosis (RVT) is associated with a doubled risk of recurrent VTE compared to those without RVT, suggesting that mechanical thrombosis removal may be warranted [37].

## 5. Inferior Vena Cava Filters and Thrombosis

The role of inferior vena cava (IVC) filters in the management of a venous thrombosis is controversial and evolving. Filter placement is currently indicated within the first four weeks, only if contraindications to AC exist, including active bleeding or recent major surgeries [26]. Filter thrombosis is a severe but rare complication. If the risk of thrombosis is high after surgery, one controversial approach dependent upon expertise is to place a retrievable filter for the high-risk period before AC therapy can be initiated safely. Specifics should be discussed with the surgeon and primary team due to the risk of significant complications with unclear long-term benefits; low retrieval rates and irregular AC therapy often lead to poor outcomes with high rates of IVC thrombosis. Active filter follow-up programs should be implemented as patients are otherwise liable to be lost to follow-up or in some cases filters are not removed at all. Successful programs report a high rate of filter retrieval, indeed as much as >95% [38–40]. Each retrievable IVC filter has a recommended dwell time, but in general IVC filters should be removed within 6 months to prevent IVC thrombosis. Gastrointestinal (GI) bleeding and intracranial hemorrhage may warrant a longer period before IVC filter removal and resumption of AC [26]. Though IVC filters have been shown to decrease the amount of PE over many years compared to AC alone, patients with filters are significantly more likely to develop DVT [41].

IVC thrombosis is a rare entity that can have dramatic consequences in morbidity and mortality and affects between 2.6 and 4% of patients with DVT [6, 42–45]. In contrast, among patients with congenital IVC abnormalities (categorized into suprarenal, renal, and infrarenal), the incidence increases to 60–80% [46–48]. Other risk factors include hypercoagulable state (thrombophilia, oral contraceptives, smoking, hormonal replacement therapy, etc.), abdominal pathologies (renal cell carcinoma, mass effect on the IVC, Budd-Chiari syndrome, etc.), and IVC filters [43, 45]. Clinical presentation includes leg heaviness, pain, swelling, and leg cramps but is highly variable based on the location, onset, and extension of clot burden.

The overlap of clinical symptoms with lower-extremity deep vein thrombosis (DVT) and its relative scarcity can make efficient diagnosis of IVC thrombosis difficult. A concise diagnostic algorithm includes risk stratification with subsequent ultrasound and venograms if indicated [43]. Sequelae of untreated IVC thrombosis include postthrombotic syndrome (PTS), shown to be as high as 90%, venous claudication in 45%, PE in 30%, and venous ulcerations in 15% of patients. Severe sequelae threatening life and limb are rare and include phlegmasia cerulea dolens and renal vein thrombosis.

Anticoagulation continues to be the cornerstone of therapy for IVC thrombosis with the goal of preventing further clot burden and facilitating the natural mechanisms of clot degradation. Multiple adjunctive therapies in the acute setting can be effective in the right clinical setting including systemic lytic therapy, catheter-directed thrombolysis, pharmacomechanical thrombectomy, aspiration thrombectomy, surgical thrombectomy, and stenting. Systemic thrombolytic therapy has shown significant short-term benefits when compared to AC therapy only including complete clot lysis of 45% compared to <5% and partial lysis of 65% compared to 20% as well as a significant reduction in PTS rates. These benefits unfortunately confer a high risk of major bleeding including intracranial hemorrhage (14% with thrombolytics versus 4% with heparin therapy) [49–52]. Alternative methods of thrombus removal are increasingly capturing these outcomes while reducing bleeding risk.

## 6. Endovascular Management of VTE

**Figure 1:**
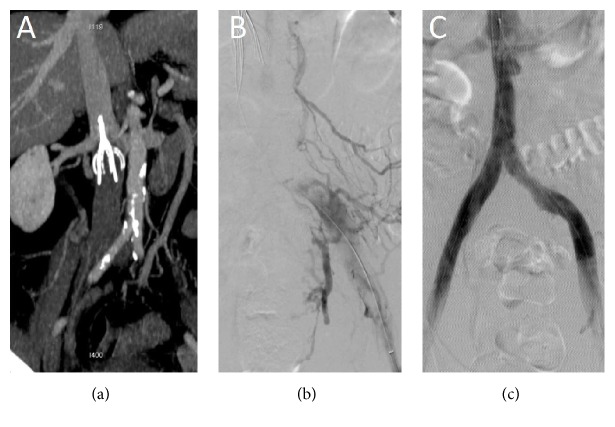
US-assisted CDT of IVC thrombosis using EKOS device. (a) Coronal reformatted contrast enhanced CT image demonstrates an IVC filter with thrombosis extending to the iliac veins. (b) Following puncture of the common femoral veins, a bilateral EKOS device was placed and 0.5 mg/hr tPA was infused for 8 hours from each groin. (c) Postvenogram demonstrates complete resolution of the thrombus with minimal thrombus at the apex of the IVC filter. This filter was subsequently removed.

Administering thrombolytic agents systemically is often associated with difficulties that include long infusion times and a high incidence of partial thrombolysis. An alternative to systemic agent administration is the use of catheter-directed thrombolytic therapy. By placing a multi-side-hole infusion catheter within the thrombus, thrombolytic agents can be administered directly in the thrombus. Catheter-directed thrombolysis (CDT) attempts to minimize the bleeding risk using smaller and focused doses of thrombolytics or using mechanical methods of clot retrieval. Endovascular techniques for thrombus removal can be found in Table 1. The use of ultrasound equipped catheters such as EkoSonic catheter (EKOS, Bothell, WA), termed US-assisted CDT, is notable as opposed to infusion-only CDT (see Figure 1). US-assisted CDT aids in dispersing the thrombolytic drug within the clot, thereby maximizing drug distribution and minimizing mechanical damage of the venous wall [10, 11, 53]. The spectrum of conditions in which CDT is applicable is broad and can include chronic iliac and/or caval stenosis or occlusions with or without IVC filter, May-Thurner syndrome and its variant, and femoropopliteal disease in addition to DVT [10, 11, 54, 55]. Yang et al. have shown that CDT also plays a role in acute superior mesenteric venous thrombosis [56]. Disadvantages of CDT include admission of the patient to an intensive care unit. Complications span a spectrum of minor bleeding at the access site to major bleeding (2.8%), PE (0.5%), and possibly significant pain and therefore it requires strict monitoring for bleeding complications and patient discomfort [10, 11]. However, if it is performed safely, some of the benefits of performing CDT can include a decreased incidence of recurrent thrombotic events with improved quality of life. Across several studies, CDT has shown the ability to achieve improved clot lysis in acute cases, resulting in improved long-term venous patency rates when compared to anticoagulation. Currently, guidelines describe in which cases CDT is suggested and include those patients whose life expectancy exceeds one year who exhibit extensive iliofemoral thrombosis, presented before 14 days after the onset of symptoms [57]. Ultimately, individuals who have long-term life expectancy are more likely to benefit due to the decreased risk of PTS and ulceration. In addition, individuals that are of working age are the most probable to benefit by undergoing the lowest risk intervention. However, oncology patients presenting a higher risk of thromboembolism must be considered and assessed before CDT given the significantly higher mortality in this group when compared to that of the general population following DVT. Postprocedure aggressive anticoagulation is advocated although this has not been well studied [10, 11]. Supportive treatments including compression stockings are also suggested [10, 11]. Finally, CDT has also not been well studied in the pediatric population but initial studies show promise. A case series on pediatric patients demonstrated effective and safe treatment of pulmonary embolism in patients aged 11–17 with no significant complications (67% complete resolution at 24 hours) [36].

### 6.1. Endovascular Management of Acute DVT and PTS

Extensive deep venous channels and their communications with the superficial venous system ensure that arterial inflow returns blood to the heart. The sentinel DVT can remain “silent” and asymptomatic in such a scenario and therefore undiagnosed until clot propagates occluding bypass channels to produce edema and pain. Venous obstruction and/or chronic insufficiency culminates in the long term resulting in PTS. However, anticoagulation treatment of a DVT at this stage is no panacea, as the age of clot is variable from region to region in the patient. A solitary acute clot is usually amenable to anticoagulation; however, risk of recurrence due to residual thrombi continues to pose a significant issue in a majority of patients [55]. Anticoagulation as monotherapy is known to lead to high rates of PTS ranging between 25% and 46% at 2 years, rising up to 90% at 5 years [55]. It has been shown that in the case of iliofemoral DVT only 30% of veins do so and that venous claudication arises in 44% of patients. Ultimately, 15% develop venous ulcer 5 years after DVT [55]. PTS is seen in 20–83% of these patients [58]. Early clot lysis has been documented with a higher likelihood of a functioning valve, while the risk of PTS is elevated by the presentation of both obstruction and reflux [58]. Several studies have indicated that anticoagulation is unlikely to be sufficient in the management of DVT: these randomized controlled studies demonstrate that systemic thrombolysis holds a significant advantage in reducing PTS versus anticoagulation monotherapy. However, major bleeding occurrences and no difference in recurrence of VTE and mortality prohibit systemic thrombolysis from becoming an acceptable standard of treatment. Subsequent percutaneous catheter and stent innovations for both arterial and venous disease have led to targeted treatment improvements which have reduced the complications encountered in systemic thrombolysis [55, 59, 60]. Targeted delivery increases drug exposure time to the actual thrombus and concomitantly limits drug exposure to that very same thrombus as compared to systemic treatment. Restitution of blood flow also leads to a cascade of further thrombus disruptions by the release of endogenous thrombolytics. CDT in conjunction with anticoagulation has been shown to have additive properties and enhanced outcomes. A systematic Cochrane review in 2004 which examined the efficacy of systemic thrombolytic therapy for acute DVT has had a recent second update in 2014 where 17 studies and 1,103 patients were included. It concluded that thrombolysis increases the patency of veins and reduces the incidence of PTS following proximal DVT by a third. CDT is now the most favored form of thrombolysis administration and there is a small increased risk of bleeding. However, protracted infusion times and high risk of bleeding complications of ~10% render systemic thrombolysis less than ideal and it is no longer in clinical use [61].

Mechanical thrombolysis (MT) and pharmacomechanical thrombolysis (PMT) have also been used for the treatment of iliofemoral DVT. These have demonstrated to be as effective as stand-alone CDT in preserving valve function and preventing PTS [62]. A Cochrane review in 2004 and 2006 concluded that “thrombolysis appears to offer advantages in terms of reducing postthrombotic syndrome and maintaining venous patency after deep vein thrombosis” [63]. In particular, PMT using recombinant tissue plasminogen activator (tPA) has shown good results with a reduction of complications such as major bleeding. Studies have also revealed that a single therapy session of CDT with MT can resolve DVT without requiring subsequent thrombolytic infusion [11, 55].

Patient selection is critical as not all patients will benefit from endovascular treatment approaches [64]. Vedantham et al. advocate a highly individualized approach to patient selection, with emphasis on clinical severity, patient preference, duration of symptoms, anatomic location of clot, generic quality of life (QOL) assessment, bleeding risk, life expectancy, and activity level [10, 11, 65, 66]. Contraindications to tPA use should not exist, as risk must not outweigh benefits; further, there must be no history of a recent cerebrovascular event, such as a transient ischemic attack, neurosurgery, or intracranial trauma and no active internal bleeding or disseminated intravascular coagulation (DIC) [10, 11, 67, 68]. Relative contraindications include, for example, recent surgery, serious allergic reaction to thrombolytic drug, contrast media or AC, pregnancy, infection, thrombocytopenia, intracranial tumor, or renal failure. Digital subtraction angiography (DSA) is utilized to determine the extent of the DVT and establish an estimate of the age of the thrombus. If patient history indicates that the thrombus is within 2 weeks old or if there is an acute thrombus on chronic setting, then CDT with tPA or CDT with MT may be appropriate [55].

The CaVenT study, carried out by Enden and colleagues, a landmark trial in 2012, published in the Lancet, investigated the efficacy of additional treatment with CDT using alteplase with the use of conventional anticoagulant treatment for acute DVT in a study [63]. A randomized trial was carried out with 209 patients and the occurrence of PTS was compared and found to be significantly lower in the group given additional treatment with CDT. Outcomes were successful with CDT: a 14.4% reduction in absolute risk in development of PTS was observed for patients treated with CDT and anticoagulation compared to anticoagulation alone at 2 years (41.1% versus 55.6% of patients), which was found to be significant (95% CI: 0.2–27.9, *p* = 0.047); this indicates an absolute risk reduction of 14% or the number needed to treat with CDT to prevent one PTS in seven patients (95% CI: 4–502) [63]. Major bleeding rate in the CDT group was 3% [63]. Despite this moderately successful result, some have commented that it in fact even underestimates the benefit of CDT and that the incidence of PTS was too high in the CDT group, hence limiting direct extrapolation of its results to clinical practice today [63]. Bækgaard conveys that CDT should not be dismissed due to these relatively mediocre results and CDT would presumably have even better results if patients were stratified in a more cogent manner [67]. Hofmann and Kuo, Sista et al., and Vedantham et al. further argue that numerous factors contributed to the modest success of CDT seen in the CaVenT study [10, 11, 69]. This includes an older drug-only CDT technique, modest patient numbers (189), and patient selection factors; that is, Enden et al. corroborate that patients with more extensive DVT and pelvic involvement were allocated to the CDT groups. These factors have been shown to be linked with higher levels of PTS. This and other caveats render this otherwise significant study lacking in some major arenas. For instance, the CDT cohort was more compliant with wearing ECSs and the proportion of patients on oral anticoagulation within the therapeutic range at follow-up was also higher. Finally, 42% of patients had adjunctive endovascular treatments including balloon angioplasty and/or stent placement. The CaVenT study has contributed to the literature, as the first prospective trial of CDT; however, subsequent further research is warranted as the findings from the CaVenT trial are quite remote from being deemed conclusive.

In another randomized single center trial, complete iliofemoral patency in over 70% of patients with CDT and only 12% with AC therapy was achieved. However, patient numbers are low (18) and follow-up is only short term at 6 months after procedure [70]. In 2014, Cakir et al. presented findings supporting the use of percutaneous aspiration thrombectomy over AC monotherapy in a randomized clinical trial involving 42 patients [71]. The TORPEDO (Thrombus Obliteration by Rapid Percutaneous Endovenous Intervention in Deep Venous Occlusion) trial devised by Sharifi et al. was a randomized controlled trial of acute symptomatic proximal DVT at a single center. The study utilized a nonvalidated PTS symptoms scale which renders its results significantly less powerful. Sharifi et al. demonstrated a 7% rate of PTS in patients treated with endovenous intervention in comparison to AC with 30% (*p* < 0.0001) at mean follow-up of 30 months [72]. Major discrepancies in measurement of clinical outcome reporting, low sample sizes, and altered treatment techniques contribute to the difficulty in guideline development and highlight the weakness of the data in the literature.

US-assisted CDT recruits the aid of an ultrasound-emitting catheter system to accelerate thrombolysis by disaggregating fibrin with the aim of improving drug access to the clot. In vitro results have been impressive; however, the results have not been replicated in patients as demonstrated by a retrospective study. The BERNUTIFUL (BERN Ultrasound-enhanced Thrombolysis for Ilio-Femoral Deep Vein Thrombosis versus Standard Catheter Directed Thrombolysis) randomized clinical trial in 2015 (recruiting 24 patients) failed to show a difference in PTS symptoms or thrombus reduction between US-assisted CDT and CDT in acute iliofemoral DVT [73]. Laiho et al. randomized 32 patients with massive iliofemoral DVT to undergo systemic thrombolysis or CDT, followed by anticoagulation. The patient sample in this study is very low; however, less reflux was seen in both deep and superficial veins, with greater preservation of valvular competence in those patients who had been treated with CDT in comparison to patients treated with systemic thrombolysis [74]. Various clinical trials have been conducted which compare CDT with adjunctive or assisting therapy such as CDT and balloon dilatation for acute IFDVT, which was unable to show a significant difference for Villalta scores between the groups [75, 76].

Results from the Dutch CAVA (CAtheter Versus Anticoagulation Alone for Acute Primary Ilio-Femoral DVT) trial are currently awaited. It assesses whether CDT therapy for the treatment of iliofemoral deep venous thrombosis (IFDVT) can reduce postthrombotic morbidity. The study population includes all consecutive patients with IFDVT presenting at centers enrolled in the trial. The incidence of PTS at one year and quality of life will be assessed at follow-up. Major bleeding during AC therapy, thrombosis recurrence, venous patency, and percentage of clot lysis after the thrombolytic procedure will be determined [77].

Another prospective, multicenter, randomized controlled study devised with funding from the National Institutes of Health is currently underway. This ongoing study which compares PMT with tPA and anticoagulation to optimal anticoagulation monotherapy in the management of acute DVT has recently completed its intake of patients. Outcomes include technical success, QOL, Villalta scale, Venous Clinical Severity Score, VTE symptoms/recurrence, major bleeding, PE, and death. Patients will be assessed every six months during a 2-year follow-up period. The Acute venous Thrombosis: Thrombus Removal with Adjunctive Catheter-directed Thrombolysis (ATTRACT) trial will help manifest CDT therapies as standard first-line medical practice in a subset of patients with acute symptomatic proximal DVT if it corroborates what many previous studies have thus far suggested [10, 11, 55].

Current well-established PTS treatment choices are limited to compression therapy, anticoagulation therapy, and endovascular or surgical approaches. No robust randomized trials have evaluated the effectiveness of procedures (such as venous bypass and endophlebectomy with reconstruction) that treat a subset of patients with severe PTS and deep venous obstruction. Stenting in inferior vena cava thrombotic obstruction and venous claudication due to venous hypertension aim for clinical benefits such as symptom relief, higher quality of life, and improved ulcer healing. Case series with a 10-year follow-up period of percutaneous endovenous stenting for chronic iliac vein outflow obstruction has indicated low morbidity, mortality, and high patency rates that corroborate the durability of the procedure in the long term. Other interventions including ablation, foam sclerotherapy, and correction of superficial venous reflux can provide benefits for PTS patients [77].

### 6.2. Endovascular Management in IVC Thrombosis and IVC Filter-Associated DVT

CDT has demonstrated effectiveness in multiple vascular territories warranting its increased use in patients with IVC thrombosis [23–25]. Utilization increased from 16% in 2005 to 35% in 2011 and complicated VTE/PE [30, 40]. Thrombosis of IVC filters is a rare complication but does occur and presents a unique challenge for CDT that is currently under study [38, 39]. A catheter-mounted balloon, an isolated-pharmacomechanical thrombolysis device (IPMTD), has been utilized in this scenario. A recent retrospective study of patients undergoing Trellis-8 Peripheral Infusion System (Covidien, Mansfield, MA) and thrombectomy, after complete IVC filter occlusion, showed that all demonstrated caval patency at a median of 7.8 months after procedure, though only 3 patients had imaging follow-up. No thromboembolic complications developed [37].

Stabilization of thrombus with fibrosis is a rapid process that can occur significantly prior to patient presentation to a hospital. To assist in removal of fibrous build-up and reduce procedure time, low-energy high-frequency ultrasound waves and physical fragmentation via rotating wires and catheters can be added to catheter interventions. These methods have been shown in observational studies to significantly reduce the incidence of PTS and quality of life [31–35].

**Figure 2:**
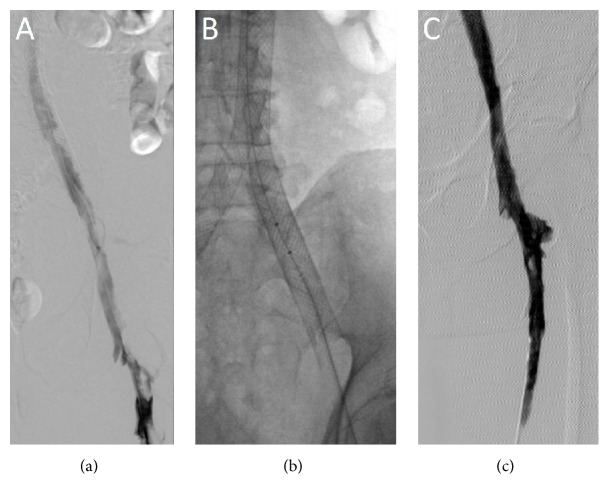
Mechanical thrombectomy of intrastent thrombosis using the AngioJet peripheral thrombectomy system. (a) Incomplete thrombosis of the IVC to iliac vein stents. (b) AngioJet thrombolysis was performed using 10 mg of tPA followed by thrombectomy. (c) Venogram reveals near-complete resolution of the thrombus.

**Figure 3:**
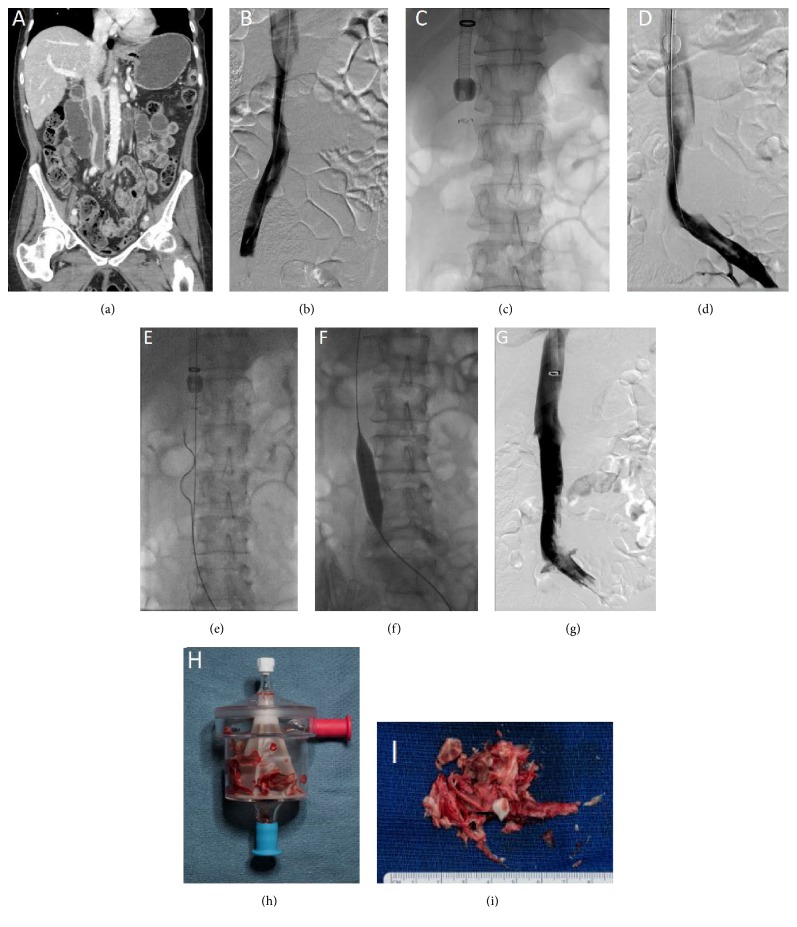
Extensive suprarenal thrombosis extending to the iliac veins treated using the AngioVac system. (a) Coronal contrast enhanced CT demonstrating the suprarenal IVC thrombosis. (b) Venogram showing the IVC thrombosis. (c) AngioVac system within the IVC engaging the IVC thrombus (d). (e) Rotational thrombectomy system is used while the AngioVac system is engaged. (f) Mechanical thrombectomy using angioplasty balloons. (g) Postprocedure venogram reveals patent infrarenal IVC and iliac veins with residual chronic thrombosis. ((h) and (i)) Aspirated predominantly chronic thrombi are shown.

Another device, the AngioJet (AngioJet Rheolytic Thrombectomy System; Medrad, Warrendale, Pennsylvania), is a pharmacomechanical action device that uses the Bernoulli principle by rapid pulses of retrograde jets for maceration and aspiration of clot contents (see Figure 2). Thrombolytic agents can be infused through the catheter to increase the clot breakdown, reduce procedure time, and promote resolution [10, 11, 66, 78]. Additionally, an alternative, a vacuum-assisted thrombectomy device, the AngioVac Cannula (AngioDynamics, Latham, New York), was designed for large vessel (IVC, pulmonary artery, etc.) thrombus removal and works through extracorporeal filtration of thrombus from venous blood while infusing the filtered blood back into the patient at a different site (see Figure 3) [12]. As thrombus removal is strictly mechanical, the AngioVac is an attractive option in patients where the bleeding risk prevents systemic thrombolytic agents. This method, however, can also be used in conjunction with thrombolytics when possible.

Patients with inferior vena cava (IVC) filter-associated DVT pose a complex clinical scenario for endovascular intervention. At present, there is limited data available to substantiate the development of a protocol. Some recent studies have attempted to deliver definitive evidence that can guide practice. Karageorgiou et al. state that they obtained restoration of flow in 87% of their patients and that 79% of the patients achieved an improvement of their presenting symptoms. The team concludes that the preexistence of an IVC filter should not be deemed as a contraindication to endovascular therapy for DVT. They do however offer caveats due to a small sample size, retrospective design, lack of a control group, lack of venographic review, and lack of long-term outcomes among numerous other limitations [79]. Further prospective studies are indeed essential. Similarly, Ganguli et al. also demonstrated good results, with no recurrence in pharmacomechanical CDT and systemic AC in treatment of lower-extremity DVT in 6 patients with atresia or agenesis of the IVC [54].

## 7. Conclusion

Venous thromboembolism remains a key healthcare concern with significant socioeconomic implications. The vascular disease, often characterized by deep venous thrombosis and pulmonary embolism, remains a major cause of mortality and morbidity. In this review, we have discussed the current understanding of the disease pathogenesis and etiology that can lead to the development and diagnosis of venous thromboembolism. We discussed how some of the current therapeutic strategies are insufficient to combat the long-term effects of the disease, including PTS and venous ulceration. Similarly, the decision to pursue inpatient versus outpatient anticoagulation treatment is essentially determined by general health, accessibility to medical care, and support at home, although other considerations are also considered. In the case of PE, echocardiography and cardiac biomarkers can suggest mortality estimates and treatment options. Next, we discussed the indications and evidence-based guidelines for inferior vena cava filters and catheter-directed thrombolysis (CDT) use and endovascular management and therapy of the disease.

Despite the progress in anticoagulation therapy and its proven ability to halt the propagation of a thrombus, it is evidently not equally adept at removing a thrombus in an afflicted area. Evidence for thrombus removal as a management component for patients with VTE has been compiled from numerous randomized trials and has shown promise. CDT can be considered a treatment approach for a cohort of PTS patients and potentially recommended for other VTE patients as well. Currently, CDT is not deemed to be a silver bullet for acute DVT but in time further subsets of patients with acute DVT may also become eligible candidates, thereby effectively sparing these patients the morbidity associated with PTS. It is hoped that this review will promote a more comprehensive review of patients with VTE by physicians as many may potentially be eligible for CDT be it assisted with MT or just AC. The ongoing ATTRACT trial is eagerly awaited as it will establish definitive guidance for near-term treatment protocols and future research directions for treatment of acute DVT. This field is at this time eminently dynamic as technological advances in devices are quickly forthcoming, while technique continues to be perfected by experienced operators. Venous interventions are sure to bring about improvements in VTE patient outcomes, and hence further trials and studies must be initiated to fully illuminate their advantages and disadvantages.

## Figures and Tables

**Table 1:** Endovascular and surgical treatment methods for thrombus removal [10–13].

Treatment modality	Description/hallmark	Prototypical example
Pharmacologic thrombolysis	Administration of thrombolytics	Catheter-based, no adjunctive mechanical assistance
*Systemic thrombolysis*	A thrombolytic is administered at an anatomic site disparate from the affected region	Intravenous catheter
*Flow-directed thrombolysis*	Intravenous catheter used to administer a thrombolytic at an anatomic site within the extremity wherein the insult has occurred; tourniquets can be used to force flow towards the DVT	Intravenous catheter and tourniquet
*Catheter-directed thrombolysis (CDT)*	Drug delivery within the thrombosed vein and US energy directed into the thrombus	Infusion catheter and US assisted catheter such as the EkoSonic catheter (EKOS, Bothell, WA)
Percutaneous mechanical thrombectomy	This modality can involve maceration, fragmentation, or aspiration; no thrombolytic is involved	Catheter-based mechanical device such as AngioVac
Pharmacomechanical CDT	Use of CDT and mechanical techniques *First generation:* can be initiated with CDT followed by mechanical technique (“infusion-first”) or vice versa (buzz-lyse) *Second generation: *simultaneous maceration and infusion of a thrombolytic	*First generation:* multiple-side hole infusion catheter *Second generation:* AngioJet, catheter-mounted balloon such as Trellis-8
Aspiration thrombectomy	Aspiration of a thrombus via a catheter using a syringe	Aspiration catheter with syringe
Balloon maceration	Utilized to fragment and disperse thrombi	Angioplasty balloon
Balloon angioplasty	Catheter-mounted balloon which supports and enlarges the venous walls	Angioplasty balloon
Stent placement	Insertion of a metallic endoprosthesis to maintain lumen patency	Stent
Surgical thrombectomy	Venotomy	Surgical instruments

## References

[1] Beckman M. G., Hooper W. C., Critchley S. E., Ortel T. L. (2010). Venous thromboembolism. A public health concern. *American Journal of Preventive Medicine*.

[2] Cohen A. T., Hamilton M., Mitchell S. A. (2015). Comparison of the novel oral anticoagulants apixaban, dabigatran, edoxaban, and rivaroxaban in the initial and long-term treatment and prevention of venous thromboembolism: Systematic review and network meta-analysis. *PLoS ONE*.

[3] Mahan C. E., Barco S., Spyropoulos A. C. (2016). Cost-of-illness model for venous thromboembolism. *Thrombosis Research*.

[4] Barco S., Woersching A. L., Spyropoulos A. C., Piovella F., Mahan C. E. (2016). European Union-28: an annualised cost-of-illness model for venous thromboembolism. *Thrombosis and Haemostasis*.

[5] Heit J. A., Silverstein M. D., Mohr D. N., Petterson T. M., O'Fallon W. M., Melton L. J. (1999). Predictors of survival after deep vein thrombosis and pulmonary embolism: a population-based, cohort study. *Archives of Internal Medicine*.

[6] White R. H. (2003). The epidemiology of venous thromboembolism. *Circulation*.

[7] Kahn S. R., Ginsberg J. S. (2002). The post-thrombotic syndrome: current knowledge, controversies, and directions for future research. *Blood Reviews*.

[8] Prandoni P., Lensing A. W. A., Cogo A. (1996). The long-term clinical course of acute deep venous thrombosis. *Annals of Internal Medicine*.

[9] MacDougall D. A., Feliu A. L., Boccuzzi S. J., Lin J. (2006). Economic burden of deep-vein thrombosis, pulmonary embolism, and post-thrombotic syndrome. *American Journal of Health-System Pharmacy*.

[10] Sista A. K., Vedantham S., Kaufman J. A., Madoff D. C. (2015). Endovascular interventions for acute and chronic lower extremity deep venous disease: state of the art. *Radiology*.

[11] Vedantham S., Sista A. K., Klein S. J. (2014). Quality improvement guidelines for the treatment of lower-extremity deep vein thrombosis with use of endovascular thrombus removal. *Journal of Vascular and Interventional Radiology*.

[12] Smith S. J., Behrens G., Sewall L. E., Sichlau M. J. (2014). Vacuum-assisted thrombectomy device (angiovac) in the management of symptomatic iliocaval thrombosis. *Journal of Vascular and Interventional Radiology*.

[13] Oklu R., Ghasemi-Rad M., Irani Z., Brinegar K. N., Toner E., Hirsch J. A. (2015). Aspiration thrombectomy using the Penumbra catheter. *Journal of Vascular and Interventional Radiology*.

[14] Kumar D. R., Hanlin E. R., Glurich I., Mazza J. J., Yale S. H. (2010). Virchow's contribution to the understanding of thrombosis and cellular biology. *Clinical Medicine & Research*.

[15] Mammen E. F. (1992). Pathogenesis of venous thrombosis. *Chest*.

[16] Nicolaides A. N., Kakkar V. V., Field E. S., Renney J. T. (1971). The origin of deep vein thrombosis: a venographic study. *British Journal of Radiology*.

[17] Aird W. C. (2007). Vascular bed-specific thrombosis. *Journal of Thrombosis and Haemostasis*.

[18] Friedman S., Beers M. B., Berkow R. (2000). Peripheral venous disease. *The Merck Manual of Geriatrics*.

[19] Mclachlin A. D., Mclachlin J. A., Jory T. A., Rawling E. G. (1960). Venous stasis in the lower extremities. *Annals of surgery*.

[20] Stein P. D., Evans H. (1967). An autopsy study of leg vein thrombosis. *Circulation*.

[21] Stamatakis J. D., Kakkar V. V., Sagar S., Lawrence D., Nairn D., Bentley P. G. (1977). Femoral vein thrombosis and total hip replacement. *British Medical Journal*.

[22] Esmon C. T. (2009). Basic mechanisms and pathogenesis of venous thrombosis. *Blood Reviews*.

[23] Hirsh J., Hull R. D., Raskob G. E. (1986). Epidemiology and pathogenesis of venous thrombosis. *Journal of the American College of Cardiology*.

[24] Wessler S., Reimer S. M., Sheps M. C. (1959). Biologic assay of a thrombosis-inducing activity in human serum. *Journal of Applied Physiology*.

[25] Cushman M., Tsai A. W., White R. H. (2004). Deep vein thrombosis and pulmonary embolism in two cohorts: the longitudinal investigation of thromboembolism etiology. *The American Journal of Medicine*.

[26] Streiff M. B., Agnelli G., Connors J. M. (2016). Guidance for the treatment of deep vein thrombosis and pulmonary embolism. *Journal of Thrombosis and Thrombolysis*.

[27] Jaff M. R., McMurtry M. S., Archer S. L. (2011). Management of massive and submassive pulmonary embolism, iliofemoral deep vein thrombosis, and chronic thromboembolic pulmonary hypertension: a scientific statement from the American Heart Association. *Circulation*.

[28] Kearon C., Akl E. A., Ornelas J. (2016). Antithrombotic therapy for VTE disease: CHEST guideline and expert panel report. *Chest*.

[29] Konstantinides S. V. (2014). 2014 ESC guidelines on the diagnosis and management of acute pulmonary embolism. *European Heart Journal*.

[30] Vedantham S., Kahn S. R., Goldhaber S. Z. (2016). Endovascular therapy for advanced post-thrombotic syndrome: proceedings from a multidisciplinary consensus panel. *Vascular Medicine*.

[31] Galanaud J.-P., Kahn S. R. (2014). Postthrombotic syndrome: a 2014 update. *Current Opinion in Cardiology*.

[32] Kahn S. R., Shrier I., Julian J. A. (2008). Determinants and time course of the postthrombotic syndrome after acute deep venous thrombosis. *Annals of Internal Medicine*.

[33] Segal J. B., Streiff M. B., Hofmann L. V., Thornton K., Bass E. B. (2007). Management of venous thromboembolism: a systematic review for a practice guideline. *Annals of Internal Medicine*.

[34] Bates S. M. (2011). Pregnancy-associated venous thromboembolism: prevention and treatment. *Seminars in Hematology*.

[35] Bates S. M., Greer A., Middeldorp S., Veenstra D. L., Prabulos A.-M., Vandvik P. O. (2012). VTE, thrombophilia, antithrombotic therapy, and pregnancy—antithrombotic therapy and prevention of thrombosis, 9th ed: American College of Chest Physicians evidence-based clinical practice guidelines. *Chest*.

[36] Lecumberri R., Alfonso A., Jiménez D. (2013). Dynamics of case-fatalilty rates of recurrent thromboembolism and major bleeding in patients treated for venous thromboembolism. *Thrombosis and Haemostasis*.

[37] Prandoni P., Lensing A. W. A., Prins M. H. (2015). The impact of residual thrombosis on the long-term outcome of patients with deep venous thrombosis treated with conventional anticoagulation. *Seminars in Thrombosis and Hemostasis*.

[38] Al-Hakim R., Kee S. T., Olinger K., Lee E. W., Moriarty J. M., McWilliams J. P. (2014). Inferior vena cava filter retrieval: effectiveness and complications of routine and advanced techniques. *Journal of Vascular and Interventional Radiology*.

[39] Alkhouli M., Morad M., Narins C. R., Raza F., Bashir R. (2016). Inferior vena cava thrombosis. *JACC Cardiovascular Interventions*.

[40] Sarosiek S., Crowther M., Sloan J. M. (2013). Indications, complications, and management of inferior vena cava filters: the experience in 952 patients at an academic hospital with a level I trauma center. *JAMA Internal Medicine*.

[41] Decousus H., Leizorovicz A., Parent F. (1998). A clinical trial of vena caval filters in the prevention of pulmonary embolism in patients with proximal deep-vein thrombosis. *New England Journal of Medicine*.

[42] Agnelli G., Verso M., Ageno W. (2008). The MASTER registry on venous thromboembolism: description of the study cohort. *Thrombosis Research*.

[43] Alkhouli M., Bashir R. (2014). Inferior vena cava filters in the United States: less is more. *International Journal of Cardiology*.

[44] Kahn S. R. (2006). The post-thrombotic syndrome: the forgotten morbidity of deep venous thrombosis. *Journal of Thrombosis and Thrombolysis*.

[45] Stein P. D., Matta F., Yaekoub A. Y. (2008). Incidence of vena cava thrombosis in the United States. *American Journal of Cardiology*.

[46] Chee Y.-L., Culligan D. J., Watson H. G. (2001). Inferior vena cava malformation as a risk factor for deep venous thrombosis in the young. *British Journal of Haematology*.

[47] Gayer G., Luboshitz J., Hertz M. (2003). Congenital anomalies of the inferior vena cava revealed on CT in patients with deep vein thrombosis. *American Journal of Roentgenology*.

[48] Sitwala P. S., Ladia V. M., Brahmbhatt P. B., Jain V., Bajaj K. (2014). Inferior vena cava anomaly: a risk for deep vein thrombosis. *North American Journal of Medical Sciences*.

[49] Arnesen H., Hoiseth A., Ly B. (1982). Streptokinase of heparin in the treatment of deep vein thrombosis. Follow-up results of a prospective study. *Acta Medica Scandinavica*.

[50] Elliot M. S., Immelman E. J., Jeffery P. (1979). A comparative randomized trial of heparin versus streptokinase in the treatment of acute proximal venous thrombosis: an interim report of a prospective trial. *British Journal of Surgery*.

[51] Neglen P., Nazzal M. M. S., Al-Hassan H. K., Christenson J. T., Eklöf B. (1992). Surgical removal of an inferior vena cava thrombus. *European Journal of Vascular Surgery*.

[52] Plate G., Eklof B., Norgren L., Ohlin P., Dahlstrom J. A. (1997). Venous thrombectomy for iliofemoral vein thrombosis—10-year Results Of A Prospective Randomised Study. *European Journal of Vascular and Endovascular Surgery*.

[53] Owens C. A. (2008). Ultrasound-enhanced thrombolysis: EKOS endo wave infusion catheter system. *Seminars in Interventional Radiology*.

[54] Ganguli S., Kalva S., Oklu R. (2012). Efficacy of lower-extremity venous thrombolysis in the setting of congenital absence or atresia of the inferior vena cava. *CardioVascular and Interventional Radiology*.

[55] Oklu R., Wicky S. (2013). Catheter-directed thrombolysis of deep venous thrombosis. *Seminars in Thrombosis and Hemostasis*.

[56] Yang S.-F., Liu B.-C., Ding W.-W., He C.-S., Wu X.-J., Li J.-S. (2014). Initial transcatheter thrombolysis for acute superior mesenteric venous thrombosis. *World Journal of Gastroenterology*.

[57] Protack C. D., Bakken A. M., Patel N., Saad W. E., Waldman D. L., Davies M. G. (2007). Long-term outcomes of catheter directed thrombolysis for lower extremity deep venous thrombosis without prophylactic inferior vena cava filter placement. *Journal of Vascular Surgery*.

[58] Grommes J., von Trotha K. T., de Wolf M. A., Jalaie H., Wittens C. H. A. (2014). Catheter-directed thrombolysis in deep vein thrombosis: Which procedural measurement predicts outcome?. *Phlebology*.

[59] Irani Z., Oklu R. (2016). The use of embolic protection device in lower extremity catheter-directed thrombolysis. *Diagnostic and Interventional Imaging*.

[60] Wicky S., Pinto E. G., Oklu R. (2013). Catheter-directed thrombolysis of arterial thrombosis. *Seminars in Thrombosis and Hemostasis*.

[61] Watson L., Broderick C., Armon M. P. (2014). Thrombolysis for acute deep vein thrombosis. *The Cochrane Database of Systematic Reviews*.

[62] Hager E., Yuo T., Avgerinos E. (2014). Anatomic and functional outcomes of pharmacomechanical and catheter-directed thrombolysis of iliofemoral deep venous thrombosis. *Journal of Vascular Surgery: Venous and Lymphatic Disorders*.

[63] Enden T., Haig Y., Kløw N.-E. (2012). Long-term outcome after additional catheter-directed thrombolysis versus standard treatment for acute iliofemoral deep vein thrombosis (the CaVenT study): a randomised controlled trial. *The Lancet*.

[64] Amin V. B., Lookstein R. A. (2014). Catheter-directed interventions for acute iliocaval deep vein thrombosis. *Techniques in Vascular and Interventional Radiology*.

[65] Vedantham S. (2011). Endovascular procedures in the management of DVT. *Hematology. American Society of Hematology. Education Program*.

[66] Vedantham S. (2015). Interventional therapy for venous thromboembolism. *Journal of Thrombosis and Haemostasis*.

[67] Bækgaard N. (2014). Benefit of catheter-directed thrombolysis for acute iliofemoral DVT: myth or reality?. *European Journal of Vascular and Endovascular Surgery*.

[68] Baekgaard N., Klitfod L., Jorgensen M. (2016). Should catheter-directed thrombolysis be monitored?. *Phlebology*.

[69] Hofmann L. V., Kuo W. T. (2012). Catheter-directed thrombolysis for acute DVT. *The Lancet*.

[70] Chen J. X., Sudheendra D., Stavropoulos S. W., Nadolski G. J. (2016). Role of catheter-directed thrombolysis in management of iliofemoral deep venous thrombosis. *RadioGraphics*.

[71] Cakir V., Gulcu A., Akay E. (2014). Use of percutaneous aspiration thrombectomy vs. anticoagulation therapy to treat acute iliofemoral venous thrombosis: 1-year follow-up results of a randomised, clinical trial. *CardioVascular and Interventional Radiology*.

[72] Sharifi M., Bay C., Mehdipour M., Sharifi J. (2012). Thrombus obliteration by rapid percutaneous endovenous intervention in deep venous occlusion (TORPEDO) trial: midterm results. *Journal of Endovascular Therapy*.

[73] Engelberger R. P., Spirk D., Willenberg T. (2015). Ultrasound-Assisted versus conventional catheter-directed thrombolysis for acute iliofemoral deep vein thrombosis. *Circulation: Cardiovascular Interventions*.

[74] Laiho M. K., Oinonen A., Sugano N. (2004). Preservation of venous valve function after catheter-directed and systemic thrombolysis for deep venous thrombosis. *European Journal of Vascular and Endovascular Surgery*.

[75] Meng Q.-Y., Li X.-Q., Jiang K. (2013). Stenting of iliac vein obstruction following catheter-directed thrombolysis in lower extremity deep vein thrombosis. *Chinese Medical Journal*.

[76] Zhang X., Ren Q., Jiang X. (2014). A prospective randomized trial of catheter-directed thrombolysis with additional balloon dilatation for iliofemoral deep venous thrombosis: a single-center experience. *CardioVascular and Interventional Radiology*.

[77] Guanella R., Kahn S. R. (2012). Post-thrombotic syndrome: current prevention and management strategies. *Expert Review of Cardiovascular Therapy*.

[78] Vedantham S. (2012). Interventional approaches to deep vein thrombosis. *American Journal of Hematology*.

[79] Karageorgiou J., Fowler K., Vedantham S., Saad N. (2016). Endovascular intervention for deep venous thrombosis in patients with inferior vena cava filters. *Vascular Medicine*.

